# An uncommon perineal embryonal rhabdomyosarcoma in adult: A case report

**DOI:** 10.1097/MD.0000000000032529

**Published:** 2022-12-30

**Authors:** Sifeng Qu, Weiwei Li, Yuan Yao, Huangwei Huang

**Affiliations:** a Medical Integration and Practice Center, Cheeloo College of Medicine, Shandong University, Jinan, China; b Department of Urology, Qilu Hospital, Cheeloo College of Medicine, Shandong University, Jinan, China; c Department of Pathology, Qilu Hospital, Cheeloo College of Medicine, Shandong University, Jinan, China; d Department of Radiology, Qilu Hospital, Cheeloo College of Medicine, Shandong University, Jinan, China.

**Keywords:** adult, case report, chemotherapy, embryonal rhabdomyosarcoma, perineum

## Abstract

Embryonal rhabdomyosarcoma (ERMS) is a major subtype of rhabdomyosarcoma, mainly affect children. There is seldom report for perineal ERMS in adults, since its rare location and the age.

**Patient concerns:**

A 20-year old male adult was admitted due to the perineal mass.

**Diagnoses:**

Diagnosis by histopathological examination of the biopsy sample was ERMS. Magnetic resonance imaging showed the tumor was found in the perineal region, with metastasis to pelvic cavity, right testis, lymph nodes and bone.

**Interventions:**

The patient received Isophosphamide and Epirubicin for 4 cycles, followed by Irinotecan and Vindesine Sulfate for 2 cycles, then cisplatin, Dacarbazine and Apatinib for 3 cycles.

**Outcome:**

The patient showed no response to chemotherapy.

**Lessons:**

Perineal ERMS in adults is very rare. There is still no standard therapy for adult ERMS. Personalized therapy might be promising treatment for each individual.

## 1. Introduction

Rhabdomyosarcoma (RMS) is an aggressive and common soft tissue sarcoma in the pediatric population. RMS cells tend to have myogenic differentiation property.^[1]^ RMS is a heterogeneous disease which contains 4 different subtypes, embryonal rhabdomyosarcoma (ERMS), alveolar rhabdomyosarcoma, pleomorphic rhabdomyosarcoma, and spindle cell/sclerosing rhabdomyosarcoma.^[2,3]^ The most common sites of RMS are head and neck, genitourinary, and extremities,^[4,5]^ while perineal and anal localizations are extremely rare and have worse prognosis.^[6,7]^

ERMS is a major subtype of RMS, the first peak of disease incidence is in early childhood, with a second peak in early adolescence.^[1]^ ERMS most commonly presents in the head and neck region and the genitourinary tract.^[4]^ The golden parameter for diagnosis of ERMS is based on histopathological feature.^[8–10]^ Immunohistochemistry staining can provide additional information for disease diagnosis. The special markers, such as Desmin, MyoD1 and Myogenin, are positive in almost all types of RMS,^[10,11]^ which could be used for differential diagnosis to exclude Ewing’s Sarcoma, granulocytic sarcoma, malignant rhabdoid tumor, and so on. Computed tomography and magnetic resonance imaging (MRI) scan are also valuable for disease evaluation, for example, tumor size, tumor location, distant metastasis, relation with neighboring tissues/organs.

## 2. Case presentation

A 20-year old man was admitted for a perineal mass at Qilu Hospital of Shandong University. The mass was noticed by the patient 1 month ago without pain. The mass was located in the perineal region and it was hard and non-movable. The maximum diameter of the mass was about 10 cm. In addition, a hard and small mass was found in the right scrotum with a maximum diameter of 2 cm. A sutured wound was found in the right inguinal region, since the patient had a biopsy of the lymph node in this region from another hospital. Written informed consent was obtained from the patient for publication of this case report and any accompanying images.

**Figure 1.**
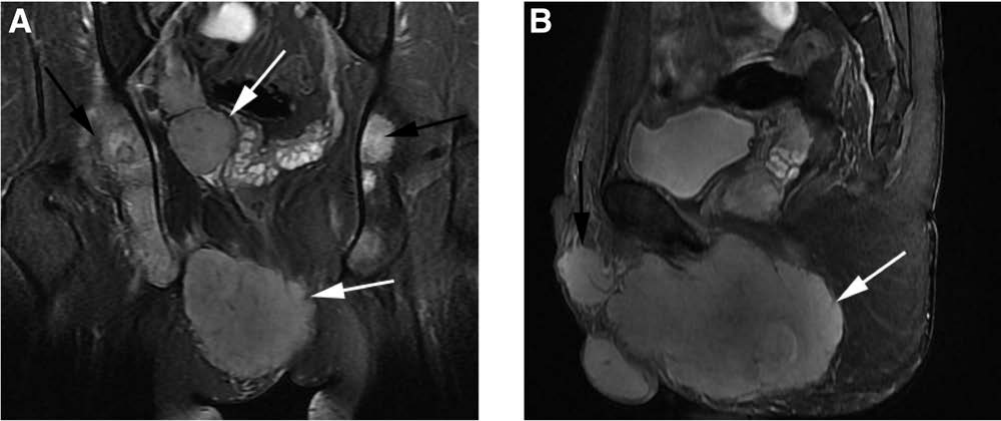
The tumor mass and metastatic sites in MRI T2WI. The tumor mass in pelvic cavity (white arrow) and metastasis in bone (black arrow) in coronal plane (A). The tumor mass in pelvic cavity (white arrow) and metastasis in right testis (black arrow) in sagittal plane (B). MRI = magnetic resonance imaging, T2WI = T2 -weighted imaging.

The MRI scan showed a large mass (11.7 cm × 7.2 cm × 8.5 cm) located on his perineum. Multiple masses were found in the pelvic cavity (right wall and floor), top of right testis and lymph nodes in bilateral inguinal regions (especially the right side). As well, scattered bone lesions were observed (Fig. 1).

**Figure 2.**
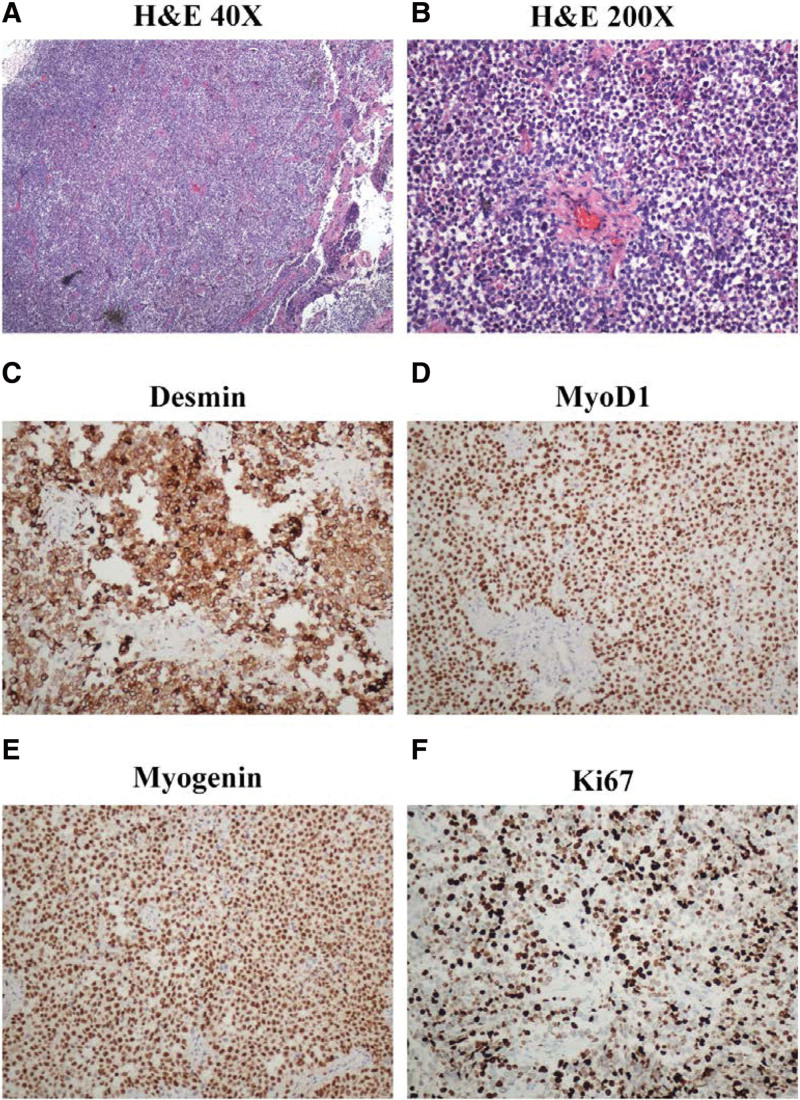
Biopsy of lymph node showed a typical ERMS pattern. H&E staining of the biopsy sample (A) (40×) and (B) (200×); IHC staining showed Desmin (+) (C), MyoD1 (+) (D), Myogenin (+) (E), and Ki67 (+) (F). ERMS = embryonal rhabdomyosarcoma, IHC = immunohistochemistry, H&E = hematoxylin and eosin.

The formalin-fixed paraffin-embedded biopsy sample was reviewed in the Department of Pathology at Qilu Hospital. The pathological diagnosis of the reviewed biopsy sample was ERMS. The hematoxylin and eosin stained formalin-fixed paraffin-embedded tissue sections showed a typical ERMS pattern: the loose and dense region containing variable condensed tumor cells, which separated by a loose, myxoid stroma may rich in connective tissue mucins (Fig. 2A and B). Immunohistochemistry staining showed the positive markers: Desmin (+), MyoD1 (+), Myogenin (+) and Ki67 (+) (Fig. 2C–F), and the negative markers: CD20 (−), CD3 (−), CD21 (−), Bcl-6 (−), MUM-1 (−), CD30 (−), S-100 (−), HMB45 (−), Melan-A (−), and SMA (−).

Considering the large tumor mass, more importantly, the multiple metastatic sites, surgical resection was not possible. The patient received chemotherapy treatment which consisted of Isophosphamide and Epirubicin for 4 cycles, followed by Irinotecan and Vindesine Sulfate for 2 cycles, followed by cisplatin, Dacarbazine and Apatinib for 3 cycles. Although the patient is alive 1 year later, but with no obvious response to treatment.

## 3. Discussion

This case is very rare due to age at occurrence and location. Although ERMS is the major subtype of RMS, the incidence of ERMS varies dramatically in different ages. The high peak of incidence is about 5 to 6 per 1000,000 between 0 and 5 years old children, while it drops to less than 1 per 1000,000 after 19-year old.^[12]^ It has been reported that metastasis raises with increasing age in ERMS,^[12]^ which is consistent with the present case. On the other hand, the most common location of ERMS include head and neck, and very few reports for ERMS in perineal region.^[13]^ Therefore, it is not common to find ERMS in an adult and located in perineal region.

As to the prognosis of ERMS, it is highly associated with disease risk stratification. Patients with distant metastasis are classified as high risk, according to the strategy defined by the Soft Tissue Sarcoma Committee of the Children’s Oncology Group.^[3,5]^ In addition, tumor size more than 5 cm, tumor located at perineal region, age more than 10-year old, positive lymph nodes are all factors with poor prognosis.^[7,13]^ Unfortunately, our patient contained all of these high risk factors, which means poor prognosis for this patient.

Chemotherapy was considered as optimal treatment for this patient. However, there is still no consensus on standard chemotherapy for adult ERMS.^[14]^ In 2017, a group of sarcoma experts was investigating to set up treatment guidelines for managing RMS. For high risk RMS patients, combined use of isophosphamide and epirubicin, or irinotecan and vindesine sulfate, are recommended.^[15]^ The current patient received multiple chemotherapy regimens, including the combinations recommended. But there was no response to treatment. Therefore, further investigation on ERMS is needed. Since next-generation sequencing is well developed and plays important role in cancer research, novel classifications of ERMS based on molecular feature might provide more information for disease management. If we can get the genomic profiling of the patient, the information can provide opportunity for him to be treated with target therapy as personalized treatment.

In conclusion, we reported a very rare case of perineal ERMS in an adult. Multiple lesions were observed in MRI image, for example, perineal region, pelvic cavity, right testis, lymph nodes and bone. Furthermore, this patient showed no response to chemotherapy. Genomic feature is needed to set up personalized treatment for adult ERMS.

## Author contributions

**Conceptualization:** Sifeng Qu.

**Data curation:** Weiwei Li, Yuan Yao, Huangwei Huang.

**Project administration:** Sifeng Qu.

**Supervision:** Sifeng Qu.

**Validation:** Weiwei Li, Yuan Yao.

**Writing – original draft:** Sifeng Qu, Weiwei Li, Yuan Yao.

**Writing – review & editing:** Sifeng Qu.
